# The Bristol Brain Health Clinic

**DOI:** 10.1002/alz.091210

**Published:** 2025-01-09

**Authors:** Anneka F Butters, Hilary Archer, Hannah Farouk, Elizabeth Coulthard

**Affiliations:** ^1^ North Bristol NHS Trust, Southmead Hospital, Bristol United Kingdom; ^2^ Bristol Medical School, University of Bristol, Bristol United Kingdom; ^3^ North Bristol NHS Trust, Bristol United Kingdom

## Abstract

**Background:**

Currently, most clinical services focus on dementia diagnosis rather than prevention, despite evidence showing benefits of preventative interventions. People often present with memory problems before having dementia, or when in the early stages and are motivated to adopt preventive lifestyle strategies to slow decline. To address gaps in clinical services we set up the Bristol Brain Health Clinic (BHC) in 2017. Here we present a clinic evaluation of diagnostic work up, interventions, and patient experience.

**Method:**

Relevant data was extracted from the medical records of patients attending The North Bristol NHS Trust BHC. Descriptive statistics were performed. Evaluation was carried out in line with European Task Force recommendations.

**Result:**

110 patients were seen in clinic between 2017‐ 2023. Mean follow‐up was 2yrs (SD 1.32). All patients had access to diagnostic assessments: neurological examination, neuropsychometry, serological and cerebrospinal fluid testing (CSF). Patients were offered bespoke lifestyle information and research opportunities.

*Patient cohort*: Mean age was 63 years (SD 11.74). 67% were male. Mean MOCA at baseline was 24 (SD 3.62).

*Dementia Risk Factors (prevalence)*: High blood pressure (49.09%), high cholesterol (47.27%), diabetic (15.45%), prediabetic (13.63%, current smoker (9.90%), ex‐smoker (39.10%), > 14 units of alcohol per week (21.81%), history of heavy drinking (14%), Mean BMI 28.01 (SD 5.85), hearing loss (40%), history of head injury (23.63%), low mood (55.45%), poor sleep quality (64%).

*CSF Studies*: 38 subjects were tested for biomarkers of neurodegenerative disease. Twenty (52.63%) were positive, 18 (47.37%) were negative.

*Diagnoses at Final Review*: Mild cognitive Impairment 27.27%, Dementia 20%, Subjective Cognitive Decline 18.91%, Uncertain 33.64%. *Aetiologies*: Alzheimer’s Disease 22.73%, Functional Cognitive Disorder 14.55%, Vascular 7.27%, Multi‐Factorial 5.45%, Parkinson’s Disease 2.73%, Lewy Body Disease 2.73, Affective Disorders 2.73%, Mixed 1.82%, Other 1.82%, Uncertain Cause 38.18%.

*Discharge*: 37.14% of the cohort were discharged with stable/resolved symptoms.

*Patient Feedback*: 84.21% ‘Extremely Likely’ to recommend our service. 92.31% felt possibly better able to cope with symptoms.

**Conclusion:**

Our population had a high prevalence of dementia risk factors and range of diagnoses. Many patients’ symptoms were stable/resolved at follow up. This population merits further investigation and continued early targeting with preventative measures.

